# Nonmodifiable Factors and Complications Contribute to Length of Stay in Robot-Assisted Partial Nephrectomy

**DOI:** 10.1089/end.2014.0424

**Published:** 2015-04-06

**Authors:** Jeffrey A. Larson, Jihad H. Kaouk, Michael D. Stifelman, Craig G. Rogers, Mohamad E. Allaf, Aaron Potretzke, Susan Marshall, Homayoun Zargar, Mark W. Ball, Sam B. Bhayani

**Affiliations:** ^1^ Division of Urology, Washington University School of Medicine, Saint Louis, Missouri.; ^2^ Cleveland Clinic, Glickman Urological and Kidney Institute, Cleveland, Ohio.; ^3^ Department of Urology, NYU Medical Center, New York, New York.; ^4^ Vattikuti Urology Institute, Henry Ford Hospital, Detroit, Michigan.; ^5^ Johns Hopkins Medical Institutions, Baltimore, Maryland.

## Abstract

***Introduction/Objective:*** Robotic-assisted partial nephrectomy (RPN) offers a mean length of stay (LOS) of 2 to 3 days. The purpose of this study is to determine the impact of modifiable and nonmodifiable risk factors on hospital LOS after RPN.

***Patients and Methods:*** We retrospectively reviewed our prospectively maintained database to identify all patients undergoing RPN for localized tumors at five US centers from 2007 to 2013. Patient and tumor characteristics were compared among hospital LOS groups. Associated factors were modeled using univariate and multivariate cumulative logistic regression to determine factors predictive of hospital LOS.

***Results:*** One thousand five hundred thirty-two patients were grouped into LOS 1 to 3 days (1298, 84.1%), LOS=4 days (133, 8.6%), and LOS >4 days (110, 7.2%). Patient demographics were similar between groups. Patients in the LOS=4 and LOS >4 day groups were more likely to have a higher Charlson comorbidity index score (mean 2.2, 3.1 and 3.8; *p*<0.001), higher nephrometry score (mean 7.1, 7.6, 7.8; *p*=0.0002), and larger tumors (mean 2.9, 3.6 and 3.5 cm; *p*<0.0001) than those in the LOS 1 to 3 day group. Significant differences in complication rates were observed when comparing LOS 1–3 (116, 8.9%), LOS=4 (40, 30%), and LOS >4 (59, 54%). According to the Clavien–Dindo classification of surgical complications, 11 grade 3 and 11 grade 4 complications occurred in patients with an LOS of 4 or more days (*p*<0.0001). Postoperative transfusion, deep vein thrombosis, pulmonary embolism, atrial fibrillation, dyspnea/atelectasis, ileus, and acute renal failure each significantly predicted a hospital LOS >4 days (*p*<0.001).

***Conclusion:*** 15.8% of patients undergoing RPN have an LOS of 4 days or more. Longer LOS was independently associated with higher Charlson index, nephrometry score (nonmodifiable factors), and perioperative complications (potentially modifiable). These data may be useful in perioperative counseling and payer precertification.

## Introduction

The incidence of small renal masses has continued to rise over the last decade, largely due to the increased detection by modern abdominal imaging. While many patients still undergo radical nephrectomy (RN), evidence supports nephron-sparing surgery, which offers favorable oncologic outcomes with preservation of renal function associated with improvement in cardiovascular morbidity and mortality.^1–5^ As such, the American Urological Association guidelines currently recommend partial nephrectomy (PN) as the standard of care for managing T1a tumors and as an alternative treatment option for T1b tumors.^6^ PN can be accomplished via a variety of modalities, including the open, laparoscopic, and robotic approaches.

While the mean hospital length of stay (LOS) for robotic-assisted partial nephrectomy (RPN) is shorter compared with open surgery, a subset of patients still requires longer hospitalizations.^7,8^ With increased scrutiny on healthcare utilization and a pressure to reduce costs, analysis of hospital LOS is essential for identifying predictors that can be used in preoperative planning and perioperative quality control and process improvement.^9^ This may become more important with the implementation of the “two-midnight rule” of Centers for Medicare & Medicaid Services, and the possibility of a specific LOS impacting reimbursement.^10^ In this context, we sought to identify and describe the characteristics present in patients requiring a longer-than-expected length of hospital stay after robotic PN.

We have previously reported our multi-institutional complication experience with RPN in 886 patients across five centers. Expansion of the series enables a more in-depth evaluation of LOS, specifically assessing factors contributing to an extended hospital LOS in a large cohort of 1532 patients who underwent robotic PN in five US centers.

## Materials and Methods

With institutional review board approval, we performed a retrospective review of de-identified, consented, prospectively maintained databases at five US centers, evaluating all patients who underwent RPN from June 2007 to August 2013. Preoperative computed tomography or magnetic resonance imaging demonstrated contrast-enhancing renal masses in all patients.

The patients were divided into three groups according to hospital LOS. The groups were chosen based on expected admission lengths. Group 1 included all patients discharged in 1 to 3 days, which is the expected LOS for RPN in published series; Group 2 included patients discharged on postoperative day (POD) 4, reflecting a slightly prolonged LOS; and Group 3 included all those who had a hospital stay of 5 or more days, reflecting a significant deviation from the expected LOS.

The RENAL nephrometry scoring system^11^ was used to stratify tumor complexity, and the Clavien system^12^ was used to grade complication severity.

All procedures were performed using the da Vinci surgical System (Intuitive, Sunnyvale, CA) through a transperitoneal or retroperitoneal approach as previously described.^13,14^ Briefly, the renal hilum is dissected, allowing individual clamping of the renal artery and vein with bulldog clamps, or a selective clamp technique. Intraoperative ultrasonography aids in identifying the tumor margin for resection. Tumor excision and sutured renorrhaphy are then performed. Similar techniques are used among all centers.

### Data collection and analysis

Staff physicians and data managers compiled data for specified patient demographics, tumor characteristics, and perioperative outcomes. Complications were recorded prospectively and classified by Clavien grade.

Hemorrhage was defined as bleeding requiring blood transfusion or therapeutic intervention.

Descriptive statistics were used to summarize patient and surgical factors. Both cumulative and multivariate logit regression models were used to test the associations of patient-related and surgical factors to the hospital LOS. SAS 9.3 software was used to perform all statistical analyses with a two-tailed *p*<0.05 considered as indicating statistical significance.

## Results

### Patient characteristics

Table 1 summarizes the clinical and pathologic characteristics, and perioperative outcomes of the 1532 patients who underwent RPN at five centers. There were 1298 patients discharged in 1 to 3 days, 133 discharged on day 4, and 110 patients discharged in 5 or more days.

**Table 1.** Comparison of Patient and Tumor Characteristics, Surgical Technique, and Perioperative Parameters in Patients Who had Hospital Admission of Less Than (Group 1), Equal to (Group 2), and Greater Than 4 Days (Group 3)

*Variables*	*Group 1* n*=1298*	*Group 2* n*=133*	*Group 3* n*=110*	p*-Value*
Patient characteristics				
Age, mean (SD), years	58.8 (12)	59.7 (13)	63.5 (11)	**0.001**
Gender, No. (%)				
Male	782 (60.7)	92 (67.1)	71 (66.3)	0.358
Female	506 (39.3)	45 (32.9)	36 (33.7)	
Ethnicity, No. (%)				0.462
Caucasian	1033	106	80	
African American	182	21	18	
Hispanic/Asian	67	16	10	
Body mass index, mean (SD) kg/m^2^	32.2 (6.8)	30.7 (7.1)	30.8 (7.2)	0.134
Age-adjusted CCI, mean (SD)	2.2 (1.7)	3.1 (1.9)	3.8 (2.3)	**<0.0001**
ASA score, mean (SD)	2.5 (0.6)	2.6 (0.6)	2.8 (0.6)	**<0.0001**
Preop eGFR, mean (SD)	86.1 (27.2)	81.2 (28.5)	74.4 (29.2)	**<0.0001**
Solitary kidney, No. (%)	26 (2.0)	11 (8.0)	5 (4.6)	0.265
Previous abdominal surgery, No. (%)	523 (40.6)	63 (46)	51 (51.4)	0.793
Tumor characteristics				
Radiographic tumor size, mean (SD) cm	2.9 (1.5)	3.6 (1.7)	3.7 (1.8)	**<0.0001**
Nephrometry score, mean (SD)	7.1 (1.2)	7.6 (1.8)	7.8 (2.0)	**0.0002**
Tumor laterality, No. (%)				
Left	381 (29.6)	73 (53.3)	52 (48.6)	0.730
Right	907 (70.4)	64 (46.7)	55 (51.4)	
Tumor location hilar, No. (%)	115 (8.9)	12 (8.7)	4 (3.7)	**0.262**
Perioperative outcomes				
Hilar clamping, No. (%)				
Unclamped	61 (4.7)	9 (6.5)	7 (6.5)	0.411
Clamped	1227 (95.3)	128 (93.5)	100 (93.5)	
WIT, mean (SD) min	18.7 (8.7)	20.8 (9.6)	23.1 (11.2)	**<0.0001**
Operative time, mean (SD), minutes	175 (55)	197 (58)	200 (63)	**<0.0001**
Blood loss, mean (SD), mL	164 (150)	286 (265)	312 (388)	**<0.0001**
Primary pathology (%)				
Benign	286 (22)	22 (16.5)	17 (15.4)	**0.0365**
Malignant	1012 (78)	111 (83.4)	93 (84.5)	
Intraoperative complications, No. (%)	14 (1)	6 (4.3)	5 (4.6)	
Postoperative complications, No. (%)				
Clavien grade ≤2	94 (7.3)	33 (24)	44 (41.1)	**<0.0001**
Clavien grade ≥3	22 (1.7)	7 (5.1)	15 (14.0)	**<0.0001**

Boldface *p*-values indicate statistical significance.

ASA=American Society of Anesthesiologists; CCI=Charlson comorbidity index; eGFR=estimated glomerular filtration rate; SD=standard deviation; WIT=warm ischemia time.

**FIG. 1.**
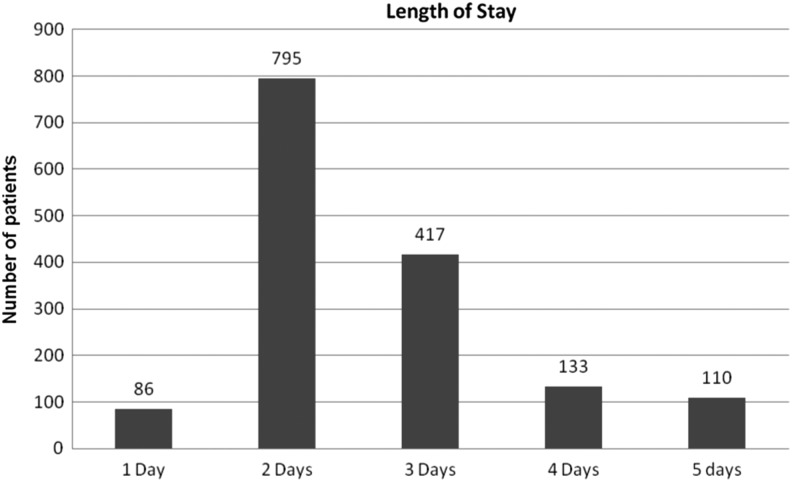
Hospital length of stay stratified by day.

Figure 1 illustrates the LOS categorized by POD. There were 86 (5.6%) patients discharged on POD 1, 795 (51.6%) on day 2, 417 (27.1%) on day 3, 133 (8.6%) on day 4, and 110 (7.2%) who stayed for 5 or more days.

### Hospital LOS predictors

On univariate analysis, lower mean age was associated with a shorter LOS (58.8 *vs* 59.7 *vs* 63.5 years; *p*<0.001). Higher median Charlson comorbidity index (CCI) was associated with a longer LOS (2.2 *vs* 3.1 *vs* 3.8, *p*<0.0001) as was a higher median American Society of Anesthesiologists (ASA) (2.5 *vs* 2.6 *vs* 2.8, *p*<0.0001). Preoperative estimated GFR was higher in patients with a low LOS (86.1 *vs* 81.2 *vs* 74.4, *p*<0.0001). Larger tumors were associated with longer LOS (2.9 *vs* 3.6 *vs* 3.7 cm, *p*<0.0001) as were more complex tumors as measured by R.E.N.A.L. nephrometry score (7.1, 7.6, 7.8, *p*=0.0002). Other patient and tumor characteristics, including gender, race, BMI, previous abdominal surgery, tumor location, and laterality, were not significantly different between the groups (all *p*>0.05).

Longer warm ischemia time (WIT) (18.7 *vs* 20.8 *vs* 23.1 minutes, *p*<0.0001), operative time (175 *vs* 197 *vs* 200 minutes, *p*<0.0001), and higher blood loss (164 *vs* 286 *vs* 312 mL, *p*<0.0001) were associated with longer hospital LOS. With regard to the type of vascular clamping (zero ischemia, artery only, and total occlusion), the three groups were similar (*p*=0.411).

After adjustment for age, gender, BMI, CCI, ASA, pathologic size, nephrometry score, laterality, clamping technique, WIT, EBL, complication, and complication Clavien grade, the variables predictive of longer LOS were age-adjusted CCI (odds ratio [OR] 2.23, 95% confidence interval [CI] 1.52–3.29, *p*<0.0001), nephrometry score (OR 1.13, 95% CI 1.01–1.26, *p*=0.041), and postoperative complications (OR 2.27, 95% CI 1.91–2.7, *p*<0.0001). Age, gender, BMI, ASA, tumor size, and WIT were not associated with LOS (Table 2).

**Table 2.** Multiple Logistic Regression Analysis Evaluating the Impact of Baseline Patient and Tumor Characteristics and Variations in Surgical Technique on Hospital Length of Stay

*Variables*	*OR (95% CI)*	p*-Value*
Patient characteristics		
Age, years	0.988 (0.97–1.06)	0.201
Gender		
Male (referent)	1.34 (0.91–1.98)	0.144
Female		
Body mass index, kg/m^2^	0.72 (0.29–1.78)	0.479
Age-adjusted CCI	2.23 (1.52–3.29)	**<0.0001**
ASA score	1.35 (0.956–1.91)	0.088
Tumor characteristics		
Pathologic size, cm	1.27 (0.81–1.99)	0.294
Nephrometry score	1.13 (1.01–1.26)	**0.041**
Tumor laterality		
Left (referent)	0.94 (0.65–1.36)	0.731
Right		
Tumor location hilar	0.94 (0.49–1.12)	0.112
Surgical technique and outcomes		
Hilar clamping		
Unclamped (referent)	1.06 (0.95–1.67)	0.427
Clamped		
WIT, minutes	1.01 (0.98–1.04)	0.327
Operative time, mean (SD), minutes	1.34 (0.69–2.82)	0.355
Blood loss, mean (SD), mL	1.54 (1.19–2.0)	**0.001**
Primary pathology		
Benign	0.79 (0.48–1.31)	0.367
Malignant		
Complication Clavien grade	2.27 (1.91–2.7)	**<0.0001**

Boldface *p-*values indicate statistical significance.

CI=confidence interval; OR=odds ratio.

Postoperative complications classified by Clavien grade and organ system are listed in Table 3. A total of 255 postoperative complications occurred in 215 patients; of these, 83 (32.5%) were classified as Clavien 1, 125 (49%) were Clavien 2, 23 (9%) were Clavien 3a, 10 (3.9%) were Clavien 3b, 13 (5.1%) were Clavien 4a, and 1 (0.4%) was Clavien 4b. There were no complication-related deaths (Clavien 5). Two patients required temporary hemodialysis for acute renal failure; no patient required permanent dialysis. Of the 14 patients with a Clavien 4 complication, 9 (65%) required a hospitalization greater than 4 days.

**Table 3.** Postoperative Complications Classified by Clavien Grade and Organ System

*Grade*	*Organ system*	*Complications*	*No. (%)*
1	Genitourinary	Acute renal failure/insufficiency (4); lymphatic leak (1); perinephric fluid (1); urinary retention (8); hematuria (5); urine leak (6)	83 (32.5)
	Cardiovascular	Atrial Fibrillation (4); tachyarrhythmia (3)	
	Pulmonary	Dyspnea (5); atelectasis (5); effusion (1)	
	Gastrointestinal	Ileus (16); diarrhea (2)	
	Dermatologic	Rash (2); ecchymosis (2); wound infection (2)	
	Psychiatric	Psychosis (2)	
	Other	Pain (3); fever (4); port site hernia (1); neuropathy (1); vertigo (1); drop in blood count not requiring intervention (8); DVT (1)	
2	Genitourinary	Bleeding requiring transfusion (47); urine leak (4); acute renal failure (3); urinary tract infection (2)	125 (49)
	Cardiovascular	Deep vein thrombosis (4); hypertensive crisis (5); tachyarrhythmia (6); atria fibrillation (10); bradycardia (1); hypotension (3)	
	Pulmonary	Pulmonary embolism (2); pneumonia (4); dyspnea (4); atelectasis (6); pulmonary edema (4); respiratory insufficiency (2)	
	Gastrointestinal	Clostridium Difficile infection (1); prolonged ileus (6)	
	Dermatologic	Wound infection (3)	
	Psychiatric	Suicidal ideation (1)	
	Other	Pain (3); incisional hernia (1); neuropathy (2); abdominal wall hematoma requiring transfusion (2)	
3a	Genitourinary	Bleeding requiring angioembolization/pseudoaneurysm (16); urine leak requiring percutaneous drainage (2)	23 (9.1)
	Cardiovascular	DVT requiring IVC filter (1)	
	Pulmonary	Pneumothorax requiring chest tube placement (2); pulmonary embolism requiring IVC filter (2)	
3b	Genitourinary	Urine leak requiring stent (7); obstructing nephrolithiasis requiring stent (1); postoperative hemorrhage requiring clot evacuation and nephrectomy (2)	10 (3.9)
4a	Genitourinary	Acute renal failure requiring hemodialysis (2); renal hemorrhage with hemodynamic instability requiring transfusion (2); Retroperitoneal hematoma requiring exploratory evacuation, transfusion (1)	13 (5.1)
	Cardiovascular	Myocardial infarction (1); delayed splenic rupture resulting in hemodynamic instability requiring operative exploration and splenectomy (1); hypertensive crisis (2); symptomatic atrial fibrillation (3); subcapsular hematoma of liver causing hemodynamic instability (1)	
	Pulmonary	Respiratory compromise secondary to subcutaneous emphysema requiring intubation (1); pulmonary embolism requiring intubation (1);	
4b	Cardiovascular	Atrial flutter associated with acute renal failure (1)	1 (0.4)

DVT=deep vein thrombosis.

With an overall complication rate of 14%, there was a significant difference between patients in the hospital for 1 to 3 days (8.9%) compared with those for 4 (30%) and 5 or more days (54%). Hemorrhagic complications were the most common, occurring almost thrice more often than cardiac complications, which were the second most prevalent type of complication in patients staying >4 days (Table 4).

**Table 4.** Postoperative Complications Stratified by Hospital Length of Stay and Organ System

*Variable*	*LOS <4 days*	*LOS=4 days*	*LOS >4*	p
Number of patients	1298	133	110	
Type of complication, *n* (%)				
Hemorrhage	26 (1.6)	17 (12.8)	28 (25.5)	**<0.001**
DVT/PE	1 (0.1)	0	7 (6.4)	**<0.001**
Cardiac	20 (1.7)	9 (7.5)	10 (9.1)	**<0.001**
Pulmonary	20 (1.6)	5 (3)	9 (8.2)	**<0.001**
GI	11 (0.9)	6 (3.8)	9 (8.2)	**<0.001**
Genitourinary	28 (2.2)	9 (9.8)	9 (8.2)	**<0.001**
Infection	7 (0.5)	0	6 (3.6)	**0.015**
Wound	10 (0.8)	0	0 (0.0)	0.577
Other	11 (0.9)	1 (0.8)	2 (2.7)	0.174

Boldface *p-*values indicate statistical significance.

LOS=length of stay.

Specific complications were analyzed for their individual effect on a hospital LOS; their incidence in patients requiring a hospital LOS greater than 4 days is listed in Table 5. There was a significant correlation between LOS >4 days and both perioperative bleeding requiring transfusion (*p*≤0.001) and identified postoperative hematoma requiring transfusion (*p*≤0.001). The occurrence of a deep vein thrombosis (DVT) was significantly correlated with LOS, as was the diagnosis of pulmonary embolism (*p*≤0.001). We found that atrial fibrillation was the most significant cardiac complication contributing to LOS (*p*≤0.001). For nonembolism pulmonary complication, dyspnea and atelectasis requiring oxygen therapy were significant (*p*≤0.001). The occurrence of ileus, instances of acute renal failure, the need for temporary hemodialysis, and the presence of postoperative fever also predicted LOS >4 days (*p*≤0.001).

**Table 5.** Postoperative Complications Classified by Type and Incidence in Length of Stay Greater Than 4 Days

*Organ system*	*Complications*	*Total No.*	*LOS >4 day (%)*	p
Hemorrhage	Bleeding requiring transfusion	50	20 (40)	**<0.001**
	Bleeding requiring angioembolization/pseudoaneurysm	16	2 (12.5)	0.134
	Postoperative hematoma/transfusion	5	5 (100)	**<0.001**
DVT/PE	DVT	4	4 (100)	**<0.001**
	DVT/Pulmonary embolism	4	3 (75)	**<0.001**
Cardiovascular	Atrial Fibrillation/flutter	18	5 (27.7)	**<0.001**
	Tachyarrhythmia	9	2 (22.2)	0.437
	Bradycardia	1	0	
	Hypertension/hypotension	10	3 (30)	**0.014**
	Myocardial infarction	1	0	
Pulmonary	Dyspnea/atelectasis	20	6 (30)	**<0.001**
	Pulmonary edema/effusion	5	1 (20)	0.267
	Pneumonia	4	0	
	Respiratory insufficiency	2	0	
	Pneumothorax requiring chest tube placement	2	1 (50)	**<0.001**
	Subcutaneous emphysema requiring intubation	1	1 (100)	**<0.001**
Gastrointestinal	Ileus	22	9 (41)	**<0.001**
	Clostridium Difficile infection/diarrhea	3	0	
	delayed splenic rupture	1	0	
Genitourinary	Acute renal failure/insufficiency	7	4 (57)	**<0.001**
	Acute renal failure requiring hemodialysis	2	2 (100)	**<0.001**
	Lymphatic leak	1	1 (100)	
	Urine leak	19	2 (10.5)	0.623
	urinary retention	8	0	
	Perinephric fluid	1	0	
	Hematuria	5	0	
	Urinary tract infection	2	0	
	obstructing nephrolithiasis requiring stent	1	0	
Dermatologic	Rash	2	0	
	Ecchymosis	2	0	
	Wound infection	5	0	
Psychiatric	Psychosis/suicidal ideation	3	0	
Other	Fever	6	6 (100)	**<0.001**
	Neuropathy	3	2 (66.7)	**<0.001**
	Pain	6	0	
	Hernia	2	0	
	Vertigo	1	0	
	Drop in blood count not requiring intervention	8	0	

Boldface *p-*values indicate statistical significance.

## Discussion

As the surgical approach to PN has evolved over the last decade, with expanding utilization of minimally invasive surgery and robotics, so have the postoperative expectations. The experience of RPN continues to mature, conferring similar or improved perioperative outcomes with excellent oncologic control compared with open surgery, and now the emphasis has begun to shift to secondary outcomes impacting patient quality of life, procedure cost, and healthcare resource utilization. Despite the advantages of minimally invasive surgery, pressures to reduce costs have led many healthcare organizations to evaluate procedure costs.

It has been suggested that robotic-assisted procedures currently cost our healthcare system more than the traditional open approach. Robotic PN has been reported to cost at least $535 to $1651 more in direct costs than the open surgical alternative, despite the shorter LOS.^15^ This estimate, similar to much of the published literature about robotic RPN, is from a high-volume center and likely an underestimate of the cost differential for many lower-volume centers, which often have longer operative times. Any increased hospital LOS, and therefore additional indirect costs after RPN, are being closely monitored and scrutinized as a potential area for cost savings. There is an emphasis on discharging patients sooner, a goal that minimally invasive surgery has largely been able to accomplish. The average LOS after RPN is 1–3 days compared with 4–8 days after an open PN.^16–18^ Clinical care pathways have been designed while emphasizing early discharge after minimally invasive PN, which have achieved discharge on POD 1 for many patients without a demonstrated increase in complications.^19^ However, even with these strategies performed in centers of excellence by high-volume surgeons, the early discharge goal is met in only 60%–75% of patients.^16,20^ In the present series, 84% of patients were discharged in 1 to 3 days, and 243 patients (16%) did not meet discharge criteria and required 4 or more days in the hospital. This demonstrates that not only will 25%–40% of patients not be able to be discharged in 1 day, but also 15% of patients will need at least 4 days to meet discharge criteria. It is, therefore, imperative that we set realistic hospital LOS expectations for our patients, other surgeons, and those involved in healthcare policy lest we establish an unrealistic standard that cannot be met in many cases.

This study has several important findings about the occurrence of prolonged hospital LOS after RPN. First are the association with LOS and the nonmodifiable risk factors of a higher CCI and more complex tumors with higher nephrometry scores. Preoperative comorbidities are a predictor of longer LOS in univariate and an independent risk factor in multivariate analysis. Comorbidities are associated with increased postoperative complications after RN,^21^ and were found to be the only predictor of an increased 30-day readmission rate after robotic PN.^22^ Patients with a higher CCI are more likely overall to have postoperative complications^23^ and as demonstrated in our analyses have a longer LOS. The R.E.N.A.L. nephrometry score is generally considered to be predictive of tumor complexity and has been significantly associated with higher incidence of postoperative complications.^24,25^ We found a significant association between nephrometry score and LOS, indicating that for larger, more complex tumors there is an increased risk of a prolonged LOS. Neither the patient's preoperative comorbidities nor tumor nephrometry score is necessarily modifiable and may indicate the need for more “work” before, during, and after surgery, despite an equivalent relative value unit reimbursement. Conceptually, this means that in an era of oversight by governmental and health insurance agencies, surgeons and healthcare systems should consider these risk factors and take a proactive approach to preventing any modifiable complications.

Second is the association with LOS and perioperative complications, a modifiable risk factor. In this series, we had an overall complication rate of 14%, which is comparable to other published reports of complication rates ranging from 0% to 20%.^26^ In our series, complications occurred in 9% of patients leaving the hospital in 1 to 3 days, whereas patients with an LOS of 4 days and 5 or more days experienced complications in 29.2% and 55.1% respectively. The severity of complication as graded by the Clavien classification was a factor in increasing LOS in both univariate and multivariate analysis. While our study illustrates that any complication regardless of severity can have a significant impact on LOS, logically the worse the complication the longer the predicted hospital LOS.

The most common complication in our series was hemorrhagic, which occurred in a total of 85 patients (5.5%), but occurred in 12.8% of patients with an LOS of 4 days and 25.5% in an LOS of 5 or more days. Of the 55 patients requiring postoperative transfusion, 45.5% stayed in the hospital longer than 4 days and were a significant contributor to LOS. In the literature, reported rates of bleeding are highly variable, ranging from 0% to 12%.^26,27^ Beyond their direct impact on patient recovery, hemorrhagic complications have a measurable secondary effect on the cost of healthcare. A previous study calculated the increased cost of having a hemorrhagic complication after LPN to be $5268.^28^ While the risk of complication is inherent to any surgical procedure, when performing an RPN, meticulous surgical technique is needed not only to avoid acute intraoperative hemorrhage but also to reduce the incidence of postoperative hemorrhagic complications that cause morbidity, prolonged LOS, and a significant increase in the cost to the healthcare system.

Our study found that development of a DVT, pulmonary embolism, and atrial fibrillation were each significant predictors of a prolonged LOS. The reported incidence of venous thromboembolism (VTE) after PN is rare, found in only 1.5% of patients undergoing radial and PN in a large retrospective analysis of 2208 patients.^29^ This study also found that a VTE event contributed significantly to a longer LOS, although it was unclear whether perioperative heparin conferred an additional benefit over pneumatic compression devices. While the incidence of VTE in our study is low, found in only 0.05% of patients, it is clear that development of a VTE has a direct impact on hospitalization. Consequently, prophylaxis should be considered in every patient.

Our findings suggest that atrial fibrillation is strongly associated with greater LOS after PN. Polanczyk et al. found that 6.1% of patients undergoing noncardiac surgery experienced an episode of atrial fibrillation, which was associated with a 33% increase in LOS.^30^ Several other studies have suggested that male gender, age >70, asthma, cardiovascular disease, ASA class 3 or greater, and intraoperative transfusion may predict postoperative arrhythmia.^31,32^ While there is no clear consensus on pharmacologic prophylaxis, identification preoperatively of high-risk patients may improve postoperative management and treatment.

This study has certain limitations. This analysis combines operative data from five large tertiary centers. While this database incorporates the early learning curves of all individual participating surgeons, the data as a whole reflect the outcomes from high-volume centers that might not be reproducible by the general urologic community. Furthermore, the referral of patients with more comorbidities and tumors with increased complexity to the tertiary care centers participating in this study introduces selection and referral bias, which may affect reported outcomes.

This study is also based on a retrospective review of a prospectively maintained database. Given the prospective data collection, the quality of the data is higher than what would be expected from a retrospective study but still lacks the integrity and accuracy of a randomized trial. There are also other variables that affect the postoperative course and LOS that were not captured in our database, such as the need for anti-coagulation, placement in extended care, social factors, weather, and travel to a distant location for recovery. Several patients at all centers traveled from outside the local community for surgery, and all surgeons were biased toward retaining some of these cases for a longer stay. Nevertheless, this large, multicenter study demonstrates appropriate perioperative outcomes for RPN; further studies evaluating how to prevent complications and a prolonged LOS are warranted.

## Conclusions

There is a significantly longer LOS in patients with the nonmodifiable risk factor of medical comorbidities, and complex tumors as well as the modifiable perioperative or postoperative complications. In an era of rising annual healthcare expenditure, where in-hospital complications and prolonged LOS are responsible for significant cost, urologists will face greater scrutiny with emphasis placed on reducing these adverse events to improve patient safety and lower costs. Despite comparable outcomes to other modalities, RPN remains a technically challenging surgery that requires significant experience in minimally invasive techniques. Long-term reduction of costs through consistently shorter, post-RPN LOS will, thus, require investment in continued training and evaluation of surgeons in this technique.

## Abbreviations Used

ASA: American Society of Anesthesiologists
CCI: Charlson comorbidity index
CI: confidence interval
DVT: deep vein thrombosis
eGFR: estimated glomerular filtration rate
LOS: length of stay
OR: odds ratio
PN: partial nephrectomy
POD: postoperative day
RN: radical nephrectomy
RPN: robotic-assisted partial nephrectomy
SD: standard deviation
VTE: venous thromboembolism
WIT: warm ischemia time
